# New Trends in the Occurrence of Yessotoxins in the Northwestern Adriatic Sea

**DOI:** 10.3390/toxins13090634

**Published:** 2021-09-09

**Authors:** Silva Rubini, Sabrina Albonetti, Simonetta Menotta, Antonio Cervo, Emanuele Callegari, Monica Cangini, Sonia Dall’Ara, Erika Baldini, Silvia Vertuani, Stefano Manfredini

**Affiliations:** 1 Experimental Zooprophylactic Institute of Lombardy and Emilia Romagna, 44124 Ferrara, Italy; silva.rubini@izsler.it (S.R.); emanuele.callegari@izsler.it (E.C.); 2 Department of Veterinary Medical Sciences, DIMEVET, University of Bologna, Via Tolara di Sopra 50, Ozzano Emilia, 40064 Bologna, Italy; sabrina.albonetti@unibo.it (S.A.); antonio.cervo@studio.unibo.it (A.C.); 3 Experimental Zooprophylactic Institute of Lombardia and Emilia Romagna, Food Chemical Department of Bologna, Via P. Fiorini 5, 40127 Bologna, Italy; simonetta.menotta@izsler.it; 4 National Reference Laboratory for Marine Biotoxins-Viale A. Vespucci, 2-47042 Cesenatico, Italy; monica.cangini@centroricerchemarine.it (M.C.); sonia.dallara@centroricerchemarine.it (S.D.); 5 Department of Life Sciences and Biotechnology, Faculty of Medicine, Pharmacy and Prevention, Master Course in Cosmetic Science, Via Fossato di Mortara 17-19, University of Ferrara, 44121 Ferrara, Italy; erika.baldini@unife.it (E.B.); smanfred@unife.it (S.M.)

**Keywords:** Yessotoxins, global warning, toxic phytoplankton, molluscs, LC-MS/MS, structure-activity relationships

## Abstract

Yessotoxins (YTXs) are polycyclic toxic ether compounds produced by phytoplanktonic dinoflagellates which accumulate in filter-feeding organisms. We know that the water temperature in our areas Northwestern Adriatic Sea is optimal for the growth of potentially toxic algae (around 20 °C). In recent years, these temperatures have remained at these levels for longer and longer periods, probably due to global warming, which has led to an excessive increase in toxin levels. The interruption of mussel harvesting caused by algae negatively affects farmers’ revenues and the availability of local fish, causing a major economic loss in Italy’s main shellfish sector. Methods: In the nine years considered, 3359 samples were examined: 1715 marine waters, 73 common clams; 732 mussels; 66 oysters; and 773 veracious clams. Bivalve molluscs were examined for the presence of marine biotoxins, including YTXs, while potentially toxic algae, including those producing YTXs, were searched for and counted in marine waters. The method adopted for the quantification of lipophilic toxins involves the use of an LC-MS/MS system. The enumeration of phytoplankton cells was performed according to the Utermhöl method. Results: Between 2012 and 2020, 706 molluscs were tested for YTXs. In total, 246 samples tested positive, i.e., 34.84%. Of the positive samples, 30 exceeded the legal limit. Conclusion: In this regard, it is essential to develop and activate, as soon as possible, an “early warning” system that allows a better control of the production areas of live bivalve molluscs, thus allowing an optimal management of the plants in these critical situations.

## 1. Introduction

Phytoplanktonic dinoflagellates produce polycyclic ether compounds, yessotoxins (YTXs), which accumulate in filter-feeding organisms [1]. The main dinoflagellate species that produce YTXs are *Protoceratium reticulatum* (Clap. and J. Lachm.) Bütschli 1885, *Lingulodinium polyedra* (F. Stein) J.D. Dodge 1989, and *Gonyaulax spinifera* (Clap. and J. Lachm.) Diesing 1866 [2,3,4]. All three species are present in the Adriatic. Actually, these algal species are widely distributed throughout the Mediterranean basin [5,6].

Since the initial discovery of YTX, many structures have been discovered and described, such as 45-hydroxyYTX, carbonYTX, 1-desulfoYTX, homoYTX, 45-hydroxyhomoYTX, and many others. More than 90 analogues have been suggested for YTXs, many of which have had their chemical structure fully identified in recent years, while others have not yet been clearly defined [7,8,9,10].

YTXs were first isolated in 1986 from scallops of the species *Patinopecten yessoensis,* a cold-water species of marine bivalve, common to the northwestern Pacific [11]. Initially, YTXs were classified in the Diarrhetic Shellfish Poison (DSP) group, both because of their liposolubility and because they were often extracted together with okaidates (okadaic acid and dinophysistoxins) when preparing extracts for mouse bioassay (MBA). Later it was found that YTXs did not cause any diarrhoeal effect when administered orally to rats [12], in contrast to okaidates and azaspiracids. 

In 2002, the European Union, through Decision 2002/225/EC, separated the marine biotoxins of the DSP complex into its four constituent categories: okadaic acid (OA) and dinophysitoxins (DTXs), yessotoxins (YTXs), pectenotoxins (PTXs) and azaspiracids (AZAs). It also established the methods of analysis and maximum permitted limits in shellfish intended for human consumption, for the different groups of lipophilic biotoxins [13].

## 2. Structure Activity Relationships

**Scheme 1:**
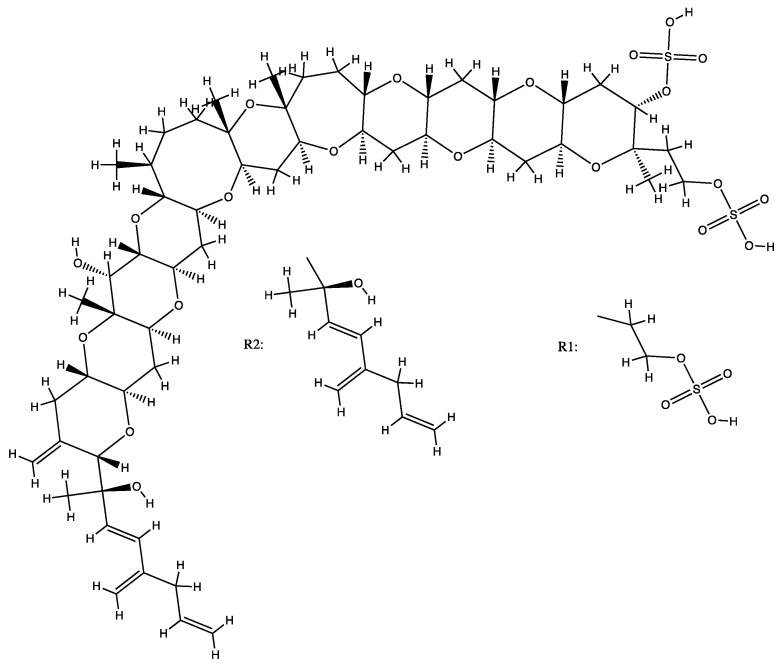
Yessotoxin-1. National Center for Biotechnology Information (2021). PubChem Compound Summary for CID 6440821, Yessotoxin. Retrieved 20 August 2021 from https://pubchem.ncbi.nlm.nih.gov/compound/Yessotoxin (accessed on 4 September 2021).

Yessotoxin (YTX) (Scheme 1) is a polycylic ether toxin carrying two substituents at the opposite side of the chain. R1 is normally an aliphatic chain bearing a second sodium sulphate salt and the R2 is a short polyenic chain or a carbonyl group. There are many structures and more certainly await to be discovered. The structure–activity relationship of yessotoxins (YTX) has been investigated and structural changes that involved the C9 terminal chain adversely affected activity, leading to the conclusion that the terminal chain is essential for the interaction of YTX in MCF-7 cells. A modulation of this terminal group lead to significant changes in toxicity [14].

The mechanism of action is still currently under investigation but point to calcium homeostasis [15].

**Scheme 2:**
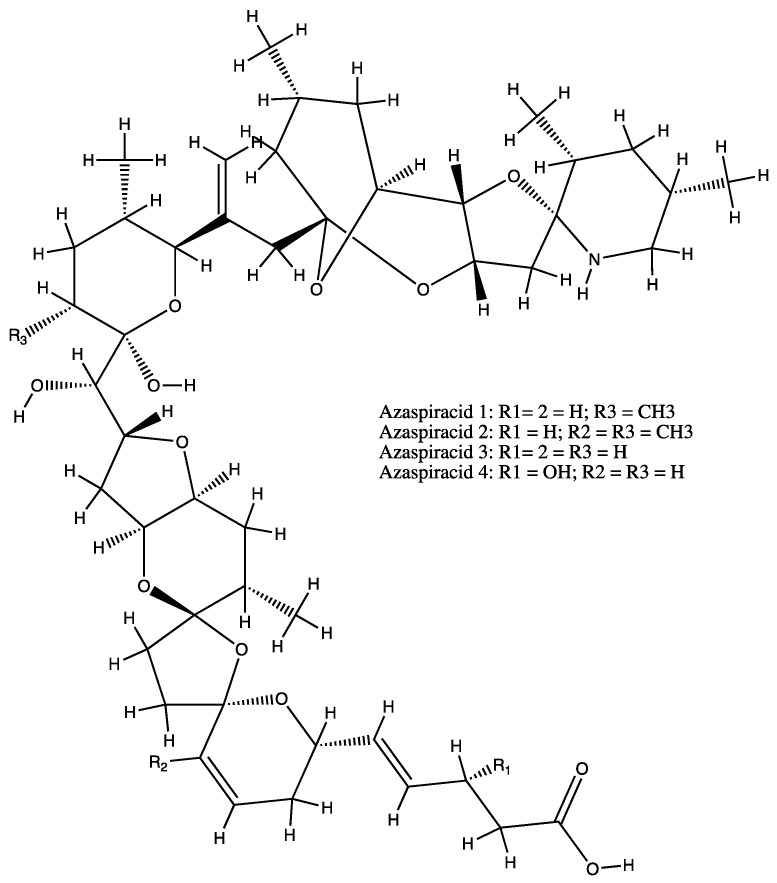
Azaspiracids (APA). National Center for Biotechnology Information (2021). PubChem Compound Summary for CID 21593892, Azaspiracid. Retrieved 20 August 2021 from https://pubchem.ncbi.nlm.nih.gov/compound/Azaspiracid (accessed on 4 September 2021).

Azaspiracids (AZA) are six-membered cyclic amine polyether toxins, featured by a unique tri-spiro-assembly and a carboxylic acid group, which gave rise to the name Azaspiracids [16] (Scheme 2). Regarding their mechanism of action, it is still under investigation but seems related to the regulation of potassium, sodium and chloride channels [17].

European legislation established until 2013, with Regulation 853/EC/2004, a maximum tolerable limit of 1 mg of yessotoxin equivalent/kg; this regulation was amended in 2013 and the tolerance limit was increased to 3.75 mg of yessotoxin equivalent/kg, with Regulation 786/EC/2013 currently in force.

Shellfish become contaminated due to their intense filtering activity. YTXs accumulate most in the hepatopancreas of molluscs, while limited absorption was found in the gastrointestinal tract. In humans, there have been no reports of adverse effects unequivocally attributable to YTXs, even when they are present in molluscs in high concentrations, but in mice, when administered intraperitoneally, they are toxic [18,19]. In particular, YTX is cardiotoxic in mice. Ferreira et al. [20] demonstrated that repeated administration of YTX caused a decrease of lymphocyte percentage and an increase of neutrophil counts, a reduction in interleukine-6 (IL-6) plasmatic levels, and histopathological splenic alterations in rats after four intraperitoneal injections of YTX at doses of 50 or 70 mg/kg that were administered every 4 d along a period of 15 days [20].

In South Africa, the mortality of millions of abalone (*Haliotis midae*) was reported in 2017 during a flowering of *Lingulodinium polyedra* and *Gonyaulax spinifera*, both of which produce YTXs [21]. However, the mechanism of action of YTXs has not yet been fully elucidated despite numerous studies [2].

YTXs have been reported worldwide [22]: Peru [9], China [10], Namibia [23], USA [8], Italy, Norway, Scotland, Japan, Chile [24], and up to the Caucasian Black Sea Coast of the Russian Federation [25].

The first positive case in molluscs in the northwestern Adriatic Sea dates back to June 1995. In fact, in the late spring/early summer of 1995, Ciminiello et al. reported, for the first time, the concomitant presence of lipophilic biotoxins of the okadaic acid group and yessotoxins in mussels from the coast of Emilia-Romagna [26]. Since then, every year the mussels have tested positive for YTXs and this positivity lasted for several months, from late summer to late autumn or even until January of the following year. The algae identified during these toxic episodes were initially *Protoceratium reticulatum* and, since 1996, *Lingulodinium polyedra*. Then, in 2004, *Gonyalux sinifera* also showed the capacity to produce YTXs on the coasts of Emilia-Romagna [5].

The main aim of the present work is to describe the trend for YTXs and analogues positivity in a high shellfish production area in the northwestern Adriatic Sea (Emilia-Romagna Region, Italy). This is in order to highlight the trend of YTXs presence from 2012 to 2020 following the replacement of the biological test (MBA) with the chemical method liquid chromatography with tandem mass spectrometry (LC/MS-MS), and to verify, thus, a new trend of the same.

At the same time, the present work also aims to highlight the prolongation over time of the presence of potentially toxic algae (whose presence, in the past, was recorded from late summer to late autumn).

## 3. Results

**Figure 1:**
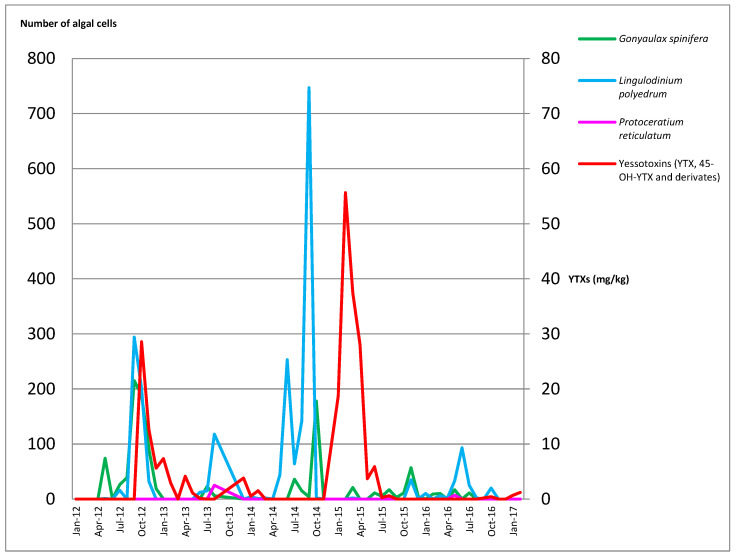
Phytoplankton numbering in water samples from the long-line sub-area in the period 2012–2017, and trends in the presence of YTXs in mussels (cells/2 mL net sample).

**Figure 2:**
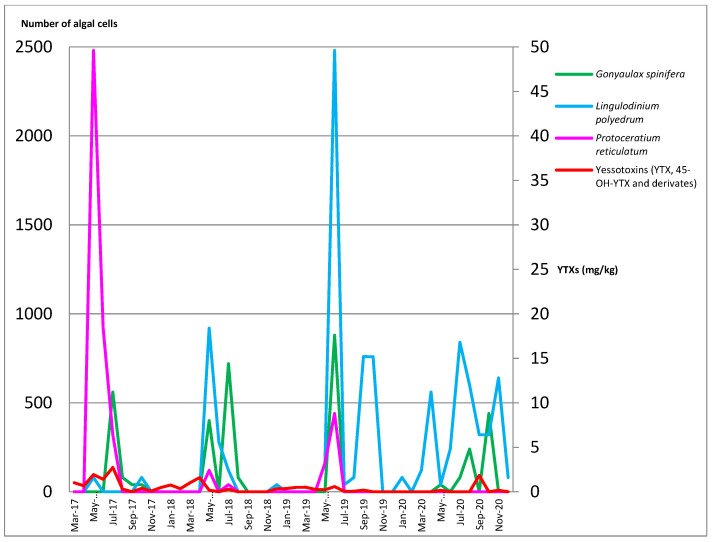
Phytoplankton numbering in water samples from the long-line sub-area in the period 2017–2020, and trends in the presence of YTXs in mussels (cells/L).

Analyses showed the presence of dinoflagellate microalgae responsible for the production of YTXs; in particular, *Lingulodinium polyedra* was the most abundant and most consistently present, *Gonyaulax spinifera* was the second most frequently reported species and, finally, *Protoceratium reticulatum* was the least frequently found species (Figure 1 and Figure 2).

YTXs were searched for in all molluscs in all sub-areas but, apart from a few cases, they were found almost exclusively in the areas furthest from the coast, known as long-lines.

The exceptions are cases where YTXs were also found in sub-areas other than the long-lines (Table 1). The two cases of mussels that tested positive for YTXs in Sacca (Case 1 and Case 5) can be attributed to the fact that fishermen sometimes leave some of the mussels they collect in long-lines for short periods in areas that are more accessible and more readily available for market requirements. The same reason is also valid for the two positive cases 7 and 8, referring to mussels harvested in the sub-area B-In. Only one case was found to be positive, but well below the legal limit, in the common clam of the sub-area called Lupini. These were three different areas (D1, D2, D3), sampled on the same day. This was the only sampling of the common clam in the whole of 2012, and several reasons, including the need for a turbo blower and the low commercial interest due to the decrease in the number of natural beds, prevented further analysis.

Finally, it is not surprising that mussels fished in the B-Out sub-area can be positive due to the coastal location, which is also subject to the influence of possible algal blooms, as is the long-line sub-area located further offshore.

The low number of positives found in areas other than the long-line allows us to exclude the sub-areas closer to the coast as areas at risk of YTXs. The situation is different for mussels farmed in the long-line. Between 2012 and 2020, 706 molluscs were tested for YTXs. In total, 246 samples tested positive, i.e., 34.84%. Of the positive samples, 30 exceeded the legal limit. This takes account of the increase in the limit itself, as decreed in Regulation 786/EC/2013. If the previous regulation were still in force and, therefore, if the maximum permitted limit were not 3.75 mg of YTXeq./Kg but 1 mg of YTXeq./Kg, the number of non-compliant molluscs would be 57, almost double the number examined between 2012 and 2020 positive for YTXs in the long-line (shown in Table 2).

### Toxicology Aspects

Cardiotoxicity in the mouse or rat and danger to humans has been demonstrated [20]; potentially toxic algae capable of producing YTXs are commonly found in the coastal waters of Emilia Romagna. In the case of these algae and toxins, it is not necessary for massive blooms to develop; on the contrary, a few hundred algal cells are sufficient to make molluscs toxic.

The water temperature in our areas is optimal for the growth of potentially toxic algae (around 20 °C) and these temperatures have been maintained at these levels for longer and longer periods, probably due to global warming. As demonstrated by Guerrini et al. [27], the production of YTXs by *P. reticulatum* is very high at 26 °C, and progressively decreased in cultures grown at 20 and 16 °C.

Chemical and physical characteristics of the Northwestern Adriatic seawaters are highly variable as the basin has a low depth and receives considerable freshwater discharge, mainly from the Po river, that significantly affects both water circulation and nutrient balance. Many studies documented the occurrence of dinoflagellate blooms after freshwater inputs following either river discharge or heavy rain falls [28,29]; recently, a significant correlation between salinity decrease and *P. reticulatum* blooms in northern Japan was reported [27].

## 4. Discussion

The health surveillance plans for molluscs, as provided for in Regulation 853/EC/2004 and subsequent amendments and integrations in the last regulation EU 2019/627, require the sampling of mussels on a weekly basis. The limit of yessotoxin equivalent/kg is currently set at 3.75 mg, which came into force in September 2013, while previously it was 1 mg/kg.

This means that the maximum toxin value was exceeded from October 2012 to January 2013, with the last exceedance in April. Even though the value set for the yessotoxin was increased, the toxicological criticality was nevertheless repeated following the accumulation of this biotoxin in mussels, with concentrations above the new limit from January to April 2015. The non-conformities recorded were managed by issuing separate bans on mussel harvesting due to yessotoxin.

It is very interesting to observe the course of algal proliferation over the years, although this has not always led to the production of biotoxins. We can ascertain a trend of seasonal growth of microalgae. They appear every year, with varying degrees of intensity, from May onwards and continue to appear until October, a sign that their multiplication is influenced by rising water temperatures.

Mussel harvesting stoppages caused by algae negatively affect farmers’ revenues and the availability of local fish, leading to a major economic loss in Italy’s leading shellfish sector.

High levels of yessotoxins are a major danger to consumers. The insidiousness lies in the mechanism of action of this toxin, as it is still unknown and does not allow us to have reliable information on the symptoms and problems that it develops in humans.

## 5. Conclusions

The data, which are recent, are intended to draw attention to the issue of harmful algal blooms (HABs), which are now constant in our seas.

YTXs first appeared on the northwest coast of the Adriatic Sea in 1995. In 2004, YTXs were detected for the first time in mussels from the northeastern Adriatic Sea. More recently, YTX has been confirmed as the most common biotoxin in the eastern-mid Adriatic Sea. Recurrence of yessotoxin (YTX) has changed the current view that DSP toxins are the main cause of shellfish toxicity in the eastern Adriatic Sea [30]. Since their appearance in the Adriatic Sea, YTXs have been consistently found in phytoplankton and bivalve molluscs. Their presence has become endemic, and we will also have to live with them in the future. Although it is still unclear what adverse effects these toxins cause in humans, YTX has been shown to display apoptotic activity in different tumour cell lines [19]; moreover, some cardiotoxic and neurological effects of YTX have been reported [20].

In this regard, it is essential to develop and activate an “early warning” system as soon as possible, which would allow better control of the production areas of live bivalve molluscs, therefore allowing optimal management of the plants in these critical situations.

Finally, regarding the production and marketing of products of animal origin, including live bivalve molluscs for human consumption, it is essential that producers comply with the indications contained in Directive 41/EC/2004, implemented by Legislative Decree No 193 of 6 November 2007.

The last two regulations to consider are number 1169/EC/2011, concerning the provision of food information to consumers (label), and Regulation 1369/EC/2017, establishing a framework for energy labelling and providing us with the expiry date, which are very important to consult when purchasing a product.

Given the topics covered in this ever-developing thesis and the increasing trend of algal blooms, simultaneous with the resulting economic issues, new insights into both algal blooms and the toxic capabilities of yessotoxins will certainly be needed in the future.

## 6. Materials and Methods

### 6.1. Study Area

The study area is located in the northwestern Adriatic Sea, between the mouth of the River Po and the mouth of the River Rhine. Data from 2011 show that almost 90,000 tonnes of shellfish are produced in this area every year [31].

**Figure 3:**
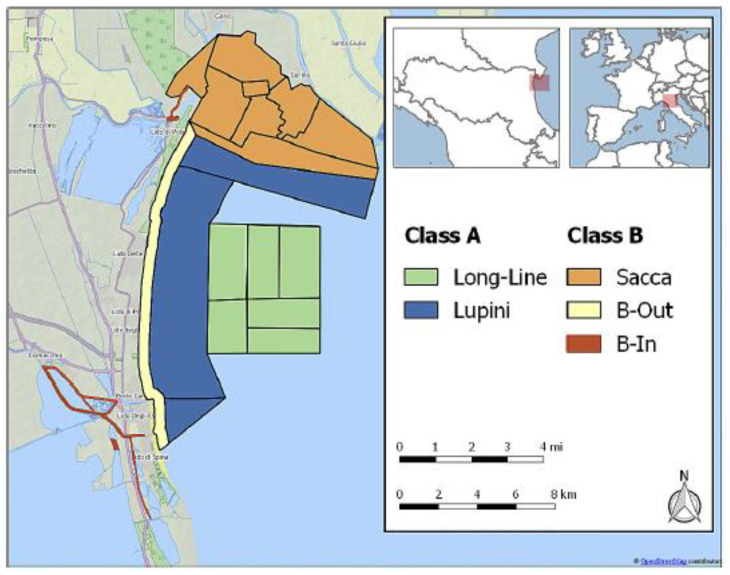
Scheme and classification of the five sub-areas in which bivalve molluscs and water samples were collected in the northwestern Adriatic Sea area, 2012–2020 [32].

**Figure 4:**
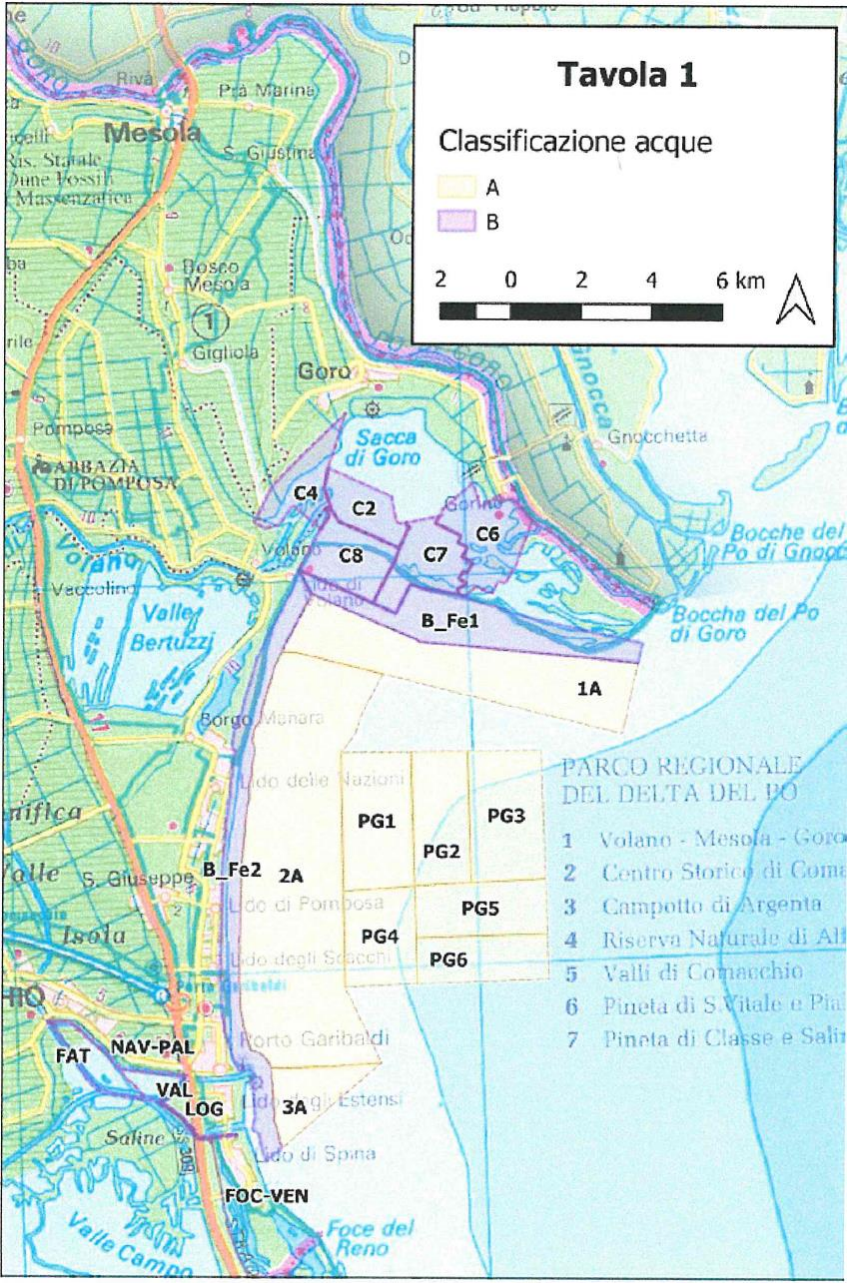
Classification of the maritime waters off the coast of Emilia-Romagna and of the regional inland waters for the breeding and harvesting of live bivalve molluscs [33].

Five sub-areas of molluscs production have been envisaged (Figure 3): (1) Long-line: the marine class A area used to breed mostly mussels (*Mytilus galloprovincialis*) and secondary oysters (*Crassostrea gigas*); (2) Lupini: the coastal marine area including seawaters between 1 and 2 nautical miles that is classified as class A area, in which natural banks of Mediterranean striped clams (*Chamelea gallina*) are present and harvested; (3) B-Out: the narrow sea coastal area and inland waters classified as class B area; (4) B-In: class B area that includes the inner channels directly connected to the sea, together with internal waters; (5) Sacca di Goro (subsequently called Sacca): the class B area included between the Po river and the marine coastline. All these last three sub-areas were used to breed Manila clams (*Tapes philippinarum*). The detail of monitoring stations are shown in Figure 4.

The Health Surveillance Plan on edible lamellibranch molluscs in Emilia-Romagna has been regularly applied since 1997, as provided for by Community legislation. The sampling frequency varies according to the mollusc species to be analysed and their filtering capacity (high in mussels, lower in clams). Depending on weather and sea conditions, mussels are sampled once a week, clams once a month, and common clams once a quarter. Until 2012, the determination of lipophilic marine biotoxins was done with the MBA, a semi-quantitative test based on the time of death of the mouse after intraperitoneal inoculation of a lipid extract of mollusc. Since March 2012, our laboratories have started to apply the chemical method in LC/MS-MS. For the present work, the results obtained in the period 2012–2020 were considered. In the nine years considered, 3359 samples were examined (Table 3): 1715 marine waters, 73 common clams (*Chamelea gallina*); 732 mussels (*Mytilus galloprovincialis*); 66 oysters (*Crassostrea gigas*); and 773 veracious clams (*Tapes philippinarum*). Bivalve molluscs were examined for the presence of marine biotoxins, including YTXs, while potentially toxic algae, including those producing YTXs, were searched for and counted in marine waters.

### 6.2. LC-MS/MS Analysis

The method adopted (EU-RL-MB, 2015) for the quantification of lipophilic toxins involves the use of an LC-MS/MS system. Toxins belonging to the YTX, AZA and PTX groups are identified and quantified by analysing a methanolic extract of the mussel homogenate, while the determination of total okadaic acid derivatives and DTXs is performed on the extract after alkaline hydrolysis. LC-MS/MS is conducted from a methanolic solution from homogenised tissue to obtain data regarding the extraction of okadaic acid, pectenotoxins, azaspiracids and yessotoxins. The extraction product was filtered and analysed by liquid chromatography combined with mass spectrometry; a fraction of this extract was subjected to alkaline hydrolysis and analysed, in order to quantify the presence of total okadaic acid derivatives.

Toxin quantification was performed by calibration of the LC-MS/MS system with standard toxin solutions of known concentration. The certified reference materials used for calibration of the system for OA, DTX1, DTX2, PTX2, YTX, homo-YTX, AZA1, AZ2, and AZA3 were purchased from the National Research Council of Canada (NRC).

The LC-MS/MS procedure was performed using an Agilent 1290 HPLC instrument coupled to an Agilent 6460 triple quadrupole, in gradient elution and negative electrospray ionization.

#### 6.2.1. Sample Preparation

Samples were stored in a refrigerator or freezer before analysis. In order to avoid toxin degradation processes, samples were analysed in the shortest time.

For successful testing, the mollusc samples must be stored in a refrigerator or freezer. At the beginning of the test, the molluscs were washed thoroughly with fresh water, paying particular attention to their exterior, and then opened by incision of the adductor muscle. The extracted pulp was left to drain and then homogenised using a blade homogeniser. The dissection steps were then carried out and the adductor muscles were then cut to open the shells. The inside of the mollusc was rinsed with fresh water to remove sand and foreign material. Finally, the flesh was removed from the shell by separating the adductor muscles and the tissue connected to the hinge.

Heat or anaesthetics should not be used before opening the shell. After removing the shells, the tissues were left to drain in a sieve to remove any remaining salt water.

For representative sampling, at least 100 to 150 g of tissue was taken and homogenised in a blade homogeniser.

#### 6.2.2. Sample Extraction and Hydrolysis

For sample extraction, 2.00 g ± 0.05 g of homogenised tissue was accurately weighed into a 50 mL centrifuge tube; then, 9.0 mL of methanol was added and the sample homogenized, using a shaker, for 3 min at maximum speed. Separation was operated with centrifuge at 2000 × *g* rotations per minute or higher for 10 min at a temperature of 20 °C, and the supernatant was then transferred into a 20 mL volumetric flask. The extraction of the residual tissue was repeated with a further 9 mL of methanol and homogenized for 10 min, again at a temperature of 20 °C, then transferred and combined with the first extract, made up to volume with 20 mL of methanol.

The extract thus obtained was analysed by LC-MS/MS to quantify the presence of toxins of the YTX, AZA, and PTX groups, while the quantification of total AO derivatives requires alkaline hydrolysis of the extract.

Hydrolysis involved adding 125 µL of 2.5 M NaOH (sodium hydroxide) to an aliquot, exactly measured, of the methanolic extract, vortexing for 30 s and heating to 76 °C for 40 min. Subsequently, the mixture was cooled to room temperature, neutralised with 125µL HCl 2.5 M (hydrochloric acid) and homogenised by vortexing for 30 s. The extract was filtered through a 0.45 µm methanol-compatible filter or through a 0.2 µm syringe filter and, depending on the sensitivity of the instrument, between 5 µL and 20 µL was injected into the mass spectrometer column.

#### 6.2.3. Working Condition of the LC-MS/MS

Reverse-phase liquid chromatography was performed using Poroshell 120 EC-C18, 2.1 × 100 mm, 2.7 µm C18 column (Agilent Technologies), and a binary pump system working at a flow rate of 0.4 mL/min with an injection volume of 10 µL. The gradient set up for the chromatographic separation used a mobile phase A (water containing 2 mM CH_3_COONH_4_ and 0.1% CH_3_COOH/MeOH; 95:5, *v*/*v*) and a mobile phase B (2 mM CH_3_COONH_4_ in MeOH). Only LC-MS grade solvents and reagents were used.

The gradient elution was as follows: with 95% A for 3 min, followed by linear gradient to 37% A over 3 min, held over 5.0 min, and within 3.0 min to 100% B, held for 2.0 min, and at least within 5.0 min back to 95% A. The run time for each analysis was 23 min.

All the mass analyses were performed on an LC-MS/MS equipped with a electrospray ionization source in the negative mode. The sample was introduced into the spray chamber using a stainless steel capillary needle; a high electrical potential was applied to generate charged ions that were directed into the mass spectrometer where they were separated and then sent to the analyser.

Analyses were carried out in multi reaction monitoring (MRM) mode with negative ionization (Table 4); selected transitions ([M-H]^−^ > [M-H-SO3]^−^: YTX *m/z* 1141.5 > 1061.5, homoYTX *m/z* 1155.5 > 1075.5, 45-OH-YXT *m/z* 1157.5 > 1077.5, 45-OH-homo-YTX *m/z* 1171.5 > 1091.5). YTX concentrations were determined by a five-point calibration curve using dilutions of a certified YTX and homo YTX standard solution, NRC, Halifax, NS, Canada). Concentrations of the YTX analogues were expressed as YTX equivalent.

### 6.3. Qualitative and Quantitative Phytoplankton Analysis

The water samples taken from 2012 to 2017 were carried out with the aid of a phytoplankton net sampler, equipped with a 20 µm mesh; from 2017 to the present, the hose-sampler has been used, i.e., a rigid tube was used, consisting of one metre segments, which were inserted into each other depending on the depth of the area to be sampled. In the Sacca farms, where the depth ranges from 1.5 to 2.5 m depending on the tidal excursion, 1-m-long tubes were used, while in the long-line farms, where the depth is about 14–16 m, 4-m-long tubes were used, i.e., tubes about as long as the mussel remains.

Within two to three hours after sampling, the water samples arrived at the laboratory where they were immediately fixed with Lugol’s fluid. The Lugol’s iodine-fixed samples were examined by inverted light microscope (Axiovert 25, Carl Zeiss International, Oberkochen, Germany). The procedure is described in the European Standard EN 15204:2006 and is based on the standard settling technique as defined by Utermöhl in 1958. It allows the estimation of the abundance and taxonomic composition of marine phytoplankton by using inverted light microscopy and sedimentation chambers.

Before pouring the samples into the sedimentation chambers, it is very important to make them homogeneous by gently inverting the bottle containing the water to be examined at least 100 times [34]. Sedimentation chambers for screened samples have a capacity of 2 mL, while sedimentation cylinders can hold 25 or 50 mL of water. The reading under the inverted light microscope was done after 15–20 min for the sedimentation chambers containing 2 mL of retinates, while for the 25 or 50 mL cylinders it is necessary to allow the phytoplankton time to settle. In the latter case, the reading can only be taken after 24 h, bearing in mind that the phytoplankton has a sedimentation time of 3–5 h per centimeter of cylinder height [29]. The results, for each algal species, were expressed as cells/2 mL when examining the net samples, and as cells/L when examining the samples collected with the hose sampler.

#### 6.3.1. Samples Collected with Plankton Net (Mesh of 20 µm) (Retinate)

For routine sampling as part of a toxin-producing phytoplankton monitoring programme, plankton nets with a mesh size of 20 µm were used. A vertical haul was taken from the depth of the mussels farm up to the surface. At the sampling site, from the boat, the plankton net was lowered; when the net reached the desired depth, it was raised slowly. Samples collected by plankton nets are inappropriate for quantitative analysis, but filtering a large amount of water can enable the early detection the toxic species even if they are in low concentrations.

#### 6.3.2. Samples Collected with Hose-Sampler (Integrated Sampling)

Hose samples are appropriate for quali-quantitative analyses of phytoplankton. The procedure used to collect the hose samples is well described in Mendez et al., 2016 [35].

## Figures and Tables

**Table 1:** Positivity cases for YTXs in sub-areas other than the long-line sub-area.

Case	Date	Molluscs Specie	Sampling Area	Sub-Area	YTXs (YTX, 45-OH-YTX and Derivates)
1	26 September 12	mussels	C1–3	Sacca	1.73 ^a^
2	25 October 12	striped clams	D3	Lupini	0.12
3	25 October 12	striped clams	D2	Lupini	0.09
4	25 October 12	striped clams	D1	Lupini	0.07
5	31 October 12	mussels	C1–3	Sacca	0.36
6	4 January 13	mussels	C9–10	B-Out	1.51 ^a^
7	28 January 13	mussels	B6	B-In	0.63
8	18 February 13	mussels	B6	B-In	2.61 ^a^
9	12 February 14	mussels	LOG	B-In	0.32
10	21 January 15	mussels	B_Fe 2	B-Out	1.18
11	27 January 15	Manila clams	VAL	B-In	0.06
12	3 October 16	mussels	B_Fe 2	B-Out	0.075
13	09 November 16	mussels	B_Fe 2	B-Out	0.077
14	25 January 17	mussels	B_Fe 2	B-Out	0.076
15	24 May 17	mussels	B_Fe 2	B-Out	0.862

^a^: values that exceeded (at that time) the legal limit (1 mg/Kg).

**Table 2:** Results of analyses carried out on mussel samples from the long-line.

		Sampling Zone						Total Sample Analysed
		PG1	PG2	PG3	PG4	PG5	PG6	
2012	NEG	16	14	14	12	14	13	83
	POS	6	6	5	4	5	4	30
Total 2012		22	20	19	16	19	17	113
2013	NEG	8	4	4	6	7	6	35
	POS	4	5	4	4	3	6	26
Total 2013		12	9	8	10	10	12	61
2014	NEG	12	9	8	9	10	12	60
	POS	2	2		1	1	1	7
Total 2014		14	11	8	10	11	13	67
2015	NEG	7	6	15	7	9	8	52
	POS	12	8	10	7	9	10	56
Total 2015		19	14	25	14	18	18	108
2016	NEG	16	15	16	17	15	15	94
	POS	1	1	1	1	2	2	8
Total 2016		17	16	17	18	17	17	102
2017	NEG	5	5	6	5	3	1	25
	POS	9	7	9	6	7	10	48
Total 2017		14	12	15	11	10	11	73
2018	NEG	7	6	8	8	6	3	38
	POS	4	4	5	4	7	8	32
Total 2018		11	10	13	12	13	11	70
2019	NEG	3	2	12	2	2	1	22
	POS	5	1	4	7	7	7	31
Total 2019		8	3	16	9	9	8	53
2020	NEG	6	5	17	10	7	6	51
	POS	2	1	1	1	2	1	8
Total 2020		8	6	18	11	9	7	59
Total 2012–2020		125	101	139	111	116	114	706

**Table 3:** Samples taken in the study area in the period from 2012 to 2020.

	Year									
Sample	2012	2013	2014	2015	2016	2017	2018	2019	2020	Total
Marine water	247	192	192	244	219	186	152	180	103	1715
Mediterranean striped clam (*Chamelea gallina*)	3	12	9	8	8	12	6	6	9	73
Mussel (*Mytilus galloprovincialis*)	140	88	71	93	105	73	66	45	51	732
Oyster (*Crassostrea gigas*)	16	5	0	16	6	3	4	8	8	66
Manila clam (*Tapes philippinarum*)	78	94	98	96	91	83	75	79	79	773
Total	484	391	370	457	429	357	303	318	250	3359

**Table 4:** Multiple reaction monitoring (MRM) acquisition parameters for YTXs [M-H]^−^.

Toxin	Prec Ion (*m/z*)	Prod Ion (*m/z*)	Frag (V)	CE (V)	ESI Mode
45-OH YTX	1157.3	1077.5	135	−37	Negative
45-OH YTX	1157.3	924.4	135	−55	Negative
45-OH YTX homo	1171.3	1091.5	135	−37	Negative
45-OH YTX homo	1171.3	938.4	135	−55	Negative
YTX	1141.3	1061.5	135	−37	Negative
YTX	1141.3	924.4	135	−55	Negative
YTX homo	1155.3	1075.5	135	−37	Negative
YTX homo	1155.3	938.4	135	−55	Negative

## Data Availability

Not applicable.
